# Assessing Knowledge, Attitudes, and Beliefs of Nurses About LGBTQ Older Adults Using a Documentary Video

**DOI:** 10.1093/geroni/igab046.740

**Published:** 2021-12-17

**Authors:** Suzanne Dutton

**Affiliations:** Sibley Memorial Hospital/Johns Hopkins, washington, District of Columbia, United States

## Abstract

Statistics reveal that lesbian, gay, bisexual, transgender, and queer (LGBTQ) older adults experience health disparities and barriers to accessing healthcare because of discrimination and fear of disclosing sexual orientation. Nurses receive limited education regarding care for LGBTQ older adults. This study exposed nurses to the documentary, Gen Silent, which details LGBTQ older adult experiences. The objective of the study was to increase participants’ understanding of LGBTQ health disparities. A one-group pre-/post-test design was conducted to test the effect of the documentary on knowledge and attitudes about LGBTQ health. A total of 379 nurses participated in the study. A questionnaire including a 16-item standardized scale and an open-ended question asking how participants would change their practice was administered before and after the intervention. Findings revealed statistically significant increases in LGBTQ knowledge and inclusive attitudes. This research supports the use of a documentary as an educational method related to LGBTQ older adults.

